# Effect of menstrual cycle on resting metabolism: A systematic review and meta-analysis

**DOI:** 10.1371/journal.pone.0236025

**Published:** 2020-07-13

**Authors:** Melissa J. Benton, Andrea M. Hutchins, J. Jay Dawes

**Affiliations:** 1 Department of Nursing, University of Colorado Colorado Springs, Colorado Springs, Colorado, United States of America; 2 Department of Human Physiology and Nutrition, University of Colorado Colorado Springs, Colorado Springs, Colorado, United States of America; 3 School of Kinesiology, Applied Health and Recreation, Oklahoma State University, Stillwater, Oklahoma, United States of America; West Virginia University, UNITED STATES

## Abstract

**Background:**

The need to control for the potential influence of menstrual cycle phase on resting metabolism (RMR) places a burden on research participants who must self-report onset of menstruation and researchers who must schedule metabolic testing accordingly.

**Purpose:**

To systematically review and analyze existing research to determine the effect of menstrual cycle on RMR.

**Methods:**

We searched PubMed, CINAHL, MEDLINE, SPORTDiscus, and Scopus databases using the search terms “menstrual cycle and metabolic rate” and “menstrual cycle and energy expenditure.” Eligibility criteria were English language, single-group repeated measures design, and RMR as either a primary or secondary outcome. Risk of bias was assessed based on study sample, measurement, and control of confounders. Differences between the follicular and luteal phases of the menstrual cycle were analyzed using the standardized mean difference in effect size.

**Results:**

Thirty English-language studies published between 1930 and December 2019 were included in the systematic review, and 26 studies involving 318 women were included in the meta-analysis. Overall, there was a small but significant effect favoring increased RMR in the luteal phase (ES = 0.33; 95% CI = 0.17, 0.49, *p* < 0.001).

**Discussion:**

Limitations include risk of bias regarding measurement of both menstrual cycle and RMR. Sample sizes were small and studies did not report control of potential confounders. Sub-group analysis demonstrated that in more recent studies published since 2000, the effect of menstrual phase was reduced and not statistically significant (ES = 0.23; 95% CI = -0.00, 0.47; *p* = 0.055). Until larger and better designed studies are available, based on our current findings, researchers should be aware of the potential confounding influence of the menstrual cycle and control for it by testing consistently in one phase of the cycle when measuring RMR in pre-menopausal women.

## Introduction

Resting metabolic rate (RMR) contributes as much as 75% to 24-hour energy expenditure [1]. As such, it plays a key role in energy balance and weight management [2]. Appropriate energy prescription to maintain energy balance over time is dependent upon accurate calculation of RMR [3], so precise measurement of metabolism is of importance to researchers. For more than 20 years, researchers have controlled for menstrual cycle fluctuations when designing studies that require measurement of RMR in young women [4–8]. Measurements have typically been restricted to the follicular phase of the menstrual cycle, which requires young women to self-report the timing and onset of menstruation to researchers. This places a burden on researchers and participants, and may create a barrier to inclusion of young, pre-menopausal women in research studies.

Data regarding the influence of menstrual cycle on metabolism are inconsistent. Although there are great intra-individual differences in RMR during the menstrual cycle, there appears to be no consistent pattern to these differences [9]. For example, early research by Bisdee and colleagues [10] provided data to suggest an effect of menstrual phase on metabolism, with RMR being lower during the follicular phase and greater in the luteal phase in a sample of 8 women. However, this was subsequently contradicted by later research conducted by Howe and colleagues [11] that reported no difference in RMR between menstrual phases in a sample of 14 women. Due to the inconsistent nature of current research and the small sample size of many of the studies, it is not possible to definitively exclude a potential confounding effect of menstrual cycle in metabolic studies of pre-menopausal women, so researchers must continue to control for menstrual cycle although it may be a needless burden. To date, the evidence regarding menstrual influences on RMR in women has not been systematically reviewed. Therefore, the purpose of this study was to systematically review and analyze existing research to determine whether the menstrual cycle influences RMR in women.

## Methods

All methods were consistent with PRISMA guidelines [12]. We did not register this review prospectively in PROSPERO.

### Search strategy

On December 18, 2018, a literature search was conducted by the first author (MJB) using the PubMed, CINAHL, MEDLINE, SPORTDiscus, and Scopus databases. No date restrictions were placed on the search. The search was updated on December 19, 2019. Search terms used were “menstrual cycle and metabolic rate” and “menstrual cycle and energy expenditure.” In addition, reference lists from relevant full-text articles were hand searched to identify any additional records that were not identified by the original electronic database search.

### Eligibility criteria

Eligibility criteria were: English language publication; single group (repeated measures) design; and measurement of RMR as either a primary or secondary outcome reported as either RMR, basal metabolic rate (BMR), sleeping metabolic rate (SMR), or excess post-exercise oxygen consumption (EPOC) in the follicular and luteal phases of the menstrual cycle. Studies that reported metabolic rate (oxygen consumption) during exercise or activities and studies not published in English were excluded. We also chose to exclude published works such as conference abstracts and graduate theses and dissertations due to concerns regarding potential bias created by low methodological quality [13].

### Study selection

All titles and abstracts were screened by the primary reviewer (MJB) to identify relevant full-text articles. Duplicates were removed by hand. Two reviewers (either AMH and MJB or JJD and MJB) then independently assessed each full-text article. Studies were included when there was agreement between both reviewers. All initial disagreements were successfully resolved by discussion between each pair of reviewers.

### Data extraction

Relevant data were identified by two reviewers (either AMH and MJB or JJD and MJB) and independently extracted by MJB. Data were extracted for first author, year of publication, geographic origin, sample size, participant age and BMI, mean follicular RMR with standard deviation or standard error, and mean luteal RMR with standard deviation or standard error, and entered into an Excel spreadsheet. BMI was derived from mean height and weight when available for individual studies that did not report BMI. After all data were entered, a second reviewer (AMH) checked accuracy.

### Quality assessment

Risk of bias in individual studies was assessed using a component approach as recommended by PRISMA guidelines [14]. A standardized assessment tool was developed based on limitations in methodology related to sample, measurement, and control of confounders. These three characteristics have been identified by the GRADE Working Group as key criteria for assessing the methodological quality and risk of bias in observational studies [15]. The assessment questions are provided in Table 1. All questions were answers as “yes” or “no.” Each study was assessed independently by two reviewers (either AMH and MJB or JJD and MJB) and risk of bias for each criterion was rated as low, moderate, or high based on the number of yes or no answers. For the study sample, risk of bias was assessed as low, moderate, or high based on cutpoints of one, two, or three “yes” responses. For both measurement and control of confounders, risk of bias was assessed as low, moderate, or high based on cutpoints of two, three, or four “yes” responses.

**Table 1:** Quality assessment questions for risk of bias based on study limitations in observational studies.

Design/Methods
**Sample**
Size justified?
Inclusion criteria clear?
Exclusion criteria clear?
**Measurement**
Conditions clearly described?
Blind assessment?
Timing/menstrual phases clearly defined?
Menstrual phases verified?
**Control of confounders**
Caffeine?
Smoking?
Medications?
Environmental temperature?
Exercise?
Other?

### Statistical analysis

Meta-analysis of pooled effect sizes was conducted using a random effects model in JASP 0.11.1 (JASP Team, University of Amsterdam, Netherlands), with the level of significance determined by *p* < 0.05 and 95% confidence intervals. Due to the variation in units of measurement across studies, differences in RMR between the follicular and luteal phases of the menstrual cycle were calculated as standardized mean differences (SMD) in effect size, and the magnitude of effect was categorized as small (≥ 0.2), medium (≥ 0.5), and large (≥ 0.8) [16]. For individual studies, the effect size for the difference between the follicular and luteal phases was calculated by subtracting the follicular mean from the luteal mean. A forest plot was generated for each analysis to illustrate the strength of the effect of menstrual cycle phase on resting metabolism. Heterogeneity between studies was assessed using *I*^2^, and values of 25%, 50%, and 75% were interpreted as indicating low, moderate, and high heterogeneity [17]. Publication bias was assessed with Egger’s test using a funnel plot for visual analysis [18]. Decisions regarding sensitivity and sub-group analyses were made post hoc and are largely exploratory. Sensitivity analysis was conducted by removing one study at a time to determine the effect of individual studies on the stability of the overall analysis, and an additional sensitivity analyses was performed to determine the effect of two studies published by the same author in consecutive years. Sub-group analyses were conducted using sample size and publication date.

When multiple time points were reported for either the follicular or luteal phases, we made an *a priori* decision to use the time points that coincided most closely with days 5–12 (follicular) and days 18–25 (luteal) of the menstrual cycle, which we interpreted to be most consistent with the majority of studies included in our analysis. When multiple conditions were reported in the same study, we prioritized RMR and BMR data because they were most commonly reported, and used SMR and EPOC data only when neither RMR nor BMR were available. Furthermore, to avoid overweighting individual studies, when multiple units of measurement were reported for the same sample, we included the effect size for only one in the analysis, with priority given to the units of measurement that were most common among studies. When standard errors were not reported for mean values, they were derived for individual studies by dividing the standard deviation by the square root of the sample size.

## Results

**Fig 1:**
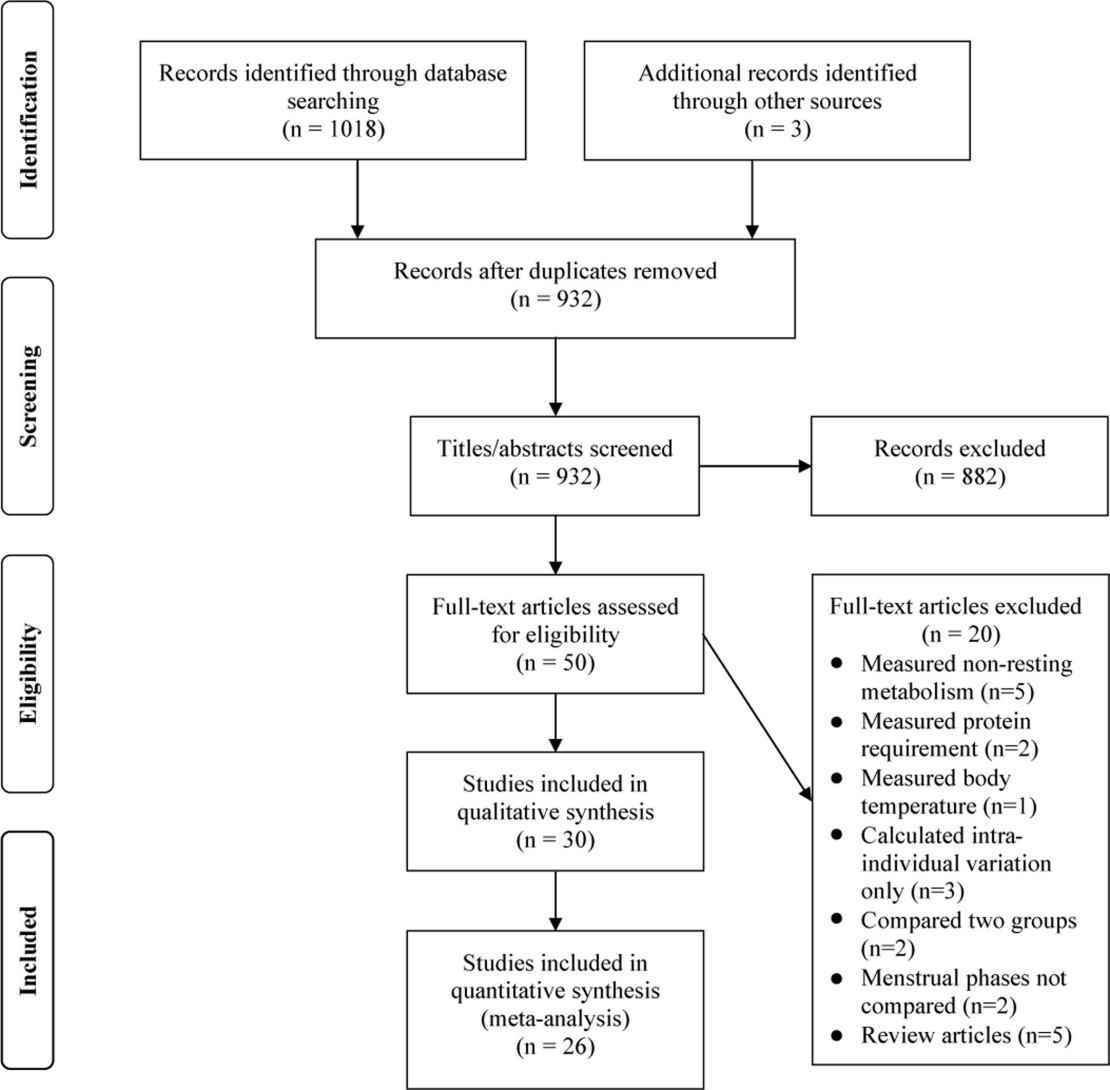
Flow diagram of selection process based on PRISMA guidelines.

In total, 1021 records were identified through the database search and hand review of reference lists that included the period from 1930 to the search date. After removal of duplicate results, 932 records were eligible for title and abstract screening. Screening resulted in 50 full-text articles for assessment (Fig 1). Thirty studies [10, 11, 19–46] were identified for inclusion in the systematic review (qualitative synthesis) that compared RMR in a single group of women during the follicular and luteal phases of the menstrual cycle. Four studies [20, 26, 27, 36] did not report mean data for metabolic rate, and due to the age of their publication dates it was not considered feasible to contact authors, so those studies were included in the systematic review only. One study [34] reported a two-group comparison (smokers vs. non-smokers), and since both groups were discrete samples, they were included as separate samples in the meta-analysis. Two studies that were published in consecutive years by the same author [30, 32] raised concern regarding potential duplication of the same sample and thus overweighting of results in the meta-analysis. Assessment of both records for inclusion criteria and measurement procedures did not support this concern and it was decided to include both sets of data in the meta-analysis. However, as described previously, sensitivity analysis was conducted with these two records removed to verify that they did not skew the results.

### Study characteristics

Study characteristics are summarized in Table 2. The majority of studies (n = 17) were published prior to the year 2000. Forty-three percent were conducted in North America (n = 13), with the remaining studies originating in Asia (n = 9), Europe (n = 7), and Australia (n = 1). Sample sizes ranged from 5 to 32 women, with the majority (n = 16) reporting samples of 10 or less women. Only 18 studies reported participant age ranges, which were between 17–47 years. By comparison, 23 studies reported mean age, while three studies did not report age at all.

**Table 2:** Study characteristics.

Year/Author	Origin	Sample	Measurements	Conditions	Time Points	Findings
1930 Conklin & McClendon [19]	United States	N = 10 Ages: 25–35 y Mean age: 22.8 y BMI: NR Inclusion: Normal women without complicating factor–menstrual pain	BMR Benedict-Roth apparatus Calculated as Calories/square meter (surface area)/hMean (SD)	Time of Day: NR Fasting: NR Temperature: NR Pre-rest: NR Duration: NR	Menstrual Postmenstrual Intermenstrual Premenstrual (not defined)	BMR tends to reach lowest level following menstruation and highest preceding menstruation
1982 Stephenson et al. [20]	United States	N = 6 Ages: 19–47 y Mean age: 26.1 y BMI: 21.5 kg/m^2^ Inclusion: Presumptively normal menstrual cycles (ranging from 28–31 d)	RMR Open circuit calorimetry Calculated as O_2_L/m Data NR	Time of Day: 7:00 am Fasting: 12–15 h Temperature: NR Pre-rest: 10 min Duration: last 5 min of 15-min rest period	Follicular (not defined) Luteal (not defined)	Oxygen uptake not statistically different during various phases of the menstrual cycle.
1988 Mehta & Pande [21]	India	N = 10 Ages: 17–22 y Mean age: NR BMI: NR Inclusion: Regular menstrual cycle	BMR (fasting and post-prandial) Closed circuit spirometry Calculated as Calories/square meter (surface area)/h Mean (SD)	Time of Day: NR Fasting: overnight followed by 5 and 15 min post consumption 200 ml milk (total of 3 measurements) Temperature: 20–25°C Pre-rest: 30 min Duration: NR	Post-menstrual (days 6–12) Pre-menstrual (days 21–27)	Fasting metabolic rate higher by 9.4% in pre-menstrual phase compared to post-menstrual. Metabolic rate following ingestion of milk not significantly different between phases.
1989 Bisdee et al. [10]	United Kingdom	N = 8 Ages: 19–32 y Mean age: 26.3 y BMI: 22.8 kg/m^2^ Inclusion: NR	BMR and SMR Whole body indirect calorimeter kJ/d Mean (SD)	BMR Time of Day: 7:00–7:30 am Fasting: 13 h Duration: 30 m SMR Time of Day: 10:00 pm-6:00 am Fasting: 4 h Duration: 8 h Temperature: 26 ± 2°C	Early follicular (onset of menses) Late follicular Early luteal (ovulation) Late luteal	Fall in energy expenditure in late follicular phase followed by rise to maximum in late luteal stage
Year/Author	Origin	Sample	Measurements	Conditions	Time Points	Findings
1991 Das & Jana [22]	India	N = 32 Age range: 17–28 y Mean age: 19.6 y BMI: 19.3 kg/m^2^ Inclusion: Fairly normal and regular menstrual history (28 ± 2 days cycle)	BMR Benedict-Roth apparatus O_2_ml/m Mean (SD)	Time of Day: NR Fasting: NR Temperature: NR Pre-rest: 30 m Duration: 7–8 m	Menstrual (days of menstrual bleeding) Follicular (days 3–4 after cessation of menses/cycle days 9–12) Luteal (cycle days 21–25)	Oxygen consumption significantly higher in luteal phase than follicular phase.
1992 Meijer et al. [23]	Netherlands	N = 16 Age range: NR Mean age: NR BMI: NR Inclusion: NR Oral contraceptives: (n = 3)	SMR Metabolic chamber kJ/m kcal/m Mean (SD)	Time of Day: 3:00–6:00 am Fasting: ≥ 9 h Temperature: NR Pre-rest: Entered chamber at 6:30 pm Duration: 3 h	Pre-ovulation (days 1–12) Post-ovulation (days 18–30)	In post-ovulation period SMR was significantly higher compared with pre-ovulation.
1993 Howe et al. [11]	United States	N = 14 Age range: 20–40 y Mean age: 31 y BMI: 24.8 kg/m^2^ Inclusion: Healthy; premenopausal	RMR Indirect calorimetry MJ/d Mean (SE)	Time of Day: 8:30 am Fasting: 12 h Temperature: NR Pre-rest: 10 m Duration: 60 m	Follicular (estradiol high/progesterone low) Luteal (progesterone high)	No significant differences among resting energy expenditures during one menstrual cycle.
1994 Lariviere et al. [24]	Canada	N = 8 Age range: 20–30 y Mean age: 24 y BMI: 20.8 kg/m^2^ Inclusion: Healthy; no history obesity or diabetes	RMR Indirect calorimetry VO_2_ml/m kJ/h Mean (SD)	Time of Day: 8:00 am Fasting: 12 h Temperature: NR Pre-rest: 30 m Duration: 60 m	Follicular (days 5–10 of menstrual cycle) Luteal (days 20–25 of menstrual cycle)	Energy expenditure was higher during the luteal phase of the cycle.
1995 Piers et al. [25]	India	N = 13 Age range: NR Mean age: 26.9 y BMI: 20.1 kg/m^2^ Inclusion: Middle or upper socioeconomic class or nonvegetarians with ad libitum food access; nonsmokers; healthy; no medications or oral contraceptives; non-pregnant; non-lactating	RMR Ventilated hood kJ/m MJ/d Mean (SD)	Time of Day: NR Fasting: NR Temperature: 24–29°C Pre-rest: 10 m Duration: 30 m	Follicular (days 6–10 of menstrual cycle) Luteal (days 21–25 of menstrual cycle)	No significant difference in RMR between follicular and luteal phases of the menstrual cycle.
Year/Author	Origin	Sample	Measurements	Conditions	Time Points	Findings
1996a Curtis et al. [26]	United Kingdom	N = 5 Age range: 19–23 y Mean age: 21.6 y BMI: 24.2 kg/m^2^ Inclusion: Taking contraceptive pill over course of one ‘menstrual cycle’	BMR Douglas bag kJ/d Data NR	Time of Day: NR Fasting: NR Temperature: NR Pre-rest: 20 m Duration: Average of two 8-m periods	Series of measurements starting on random day of cycle and made on most weekdays over a period of five consecutive weeks	BMR during early part of the cycle not significantly different from later part of the cycle.
1996b Curtis et al. [27]	United Kingdom	N = 12 Age range: 20–35 y Mean age: 22.5 y BMI: 22.5 kg/m^2^ Inclusion: Non-smokers; no use of contraceptive pills; normal menstrual cycle.	BMR Douglas bag (n = 6) Indirect calorimetry (n = 6) Data NR	Time of Day: All measurements completed by mid-morning Fasting: Usually 12–14 h Temperature: 24–26°C Pre-rest: NR Duration: NR	Menstrual cycle normalized from first day of menstrual period to the day before beginning of next period. Repeated measurements for at least one complete menstrual cycle.	BMR during early follicular phase significantly lower than late luteal phase.
1996 Melanson et al. [28]	United States	N = 8 Age range: NR Mean age: NR BMI: NR Inclusion: Normally menstruating; good health; normal glucose tolerance test; non-smokers; no oral contraceptives or other medications; moderate consumption of caffeine and alcohol.	RMR Indirect calorimetry kJ/d Mean (SE)	Time of Day: Morning Fasting: 12 h Temperature: “Thermoneutral” Pre-rest: NR Duration: 40 m	Follicular (days 6–11 of menstrual cycle) Luteal (days 16–26 of menstrual cycle)	Luteal RMR was significantly higher than in the follicular phase.
Year/Author	Origin	Sample	Measurements	Conditions	Time Points	Findings
1997 Tai et al. [29]	United States	N = 8 Age range: 22–38 y Mean age: 27.9 y BMI: 21.3 kg/m^2^ Inclusion: Not pregnant or breast-feeding; within 5% desirable body weight; free of known illness or gynecologic problems; normal menstrual cycles over previous year; no oral contraceptives or other drugs; stable body weight with no dieting during previous 6 months; non-smokers; no physical training for previous 6 months, no regular exercise during study period.	RMR Metabolic cart kJ/m Mean (SE)	Time of Day: Morning Fasting: 10–12 h Temperature: 22–25°C Pre-rest: 40 m Duration: Average of three 7-m periods	Postmenstrual/early follicular (days 2–4 after menstruation began) Follicular (days 7–10) Luteal (days 19–22) Premenstrual/late luteal (days 25–28)	No significant differences in RMR among four phases of the menstrual cycle.
1998 Matsuo et al. [30]	Japan	N = 9 Age range: 18–19 y Mean age: 18.7 y BMI: 20.6 kg/m^2^ Inclusion: Healthy; free of disease; normal menstrual cycle; no habit of daily exercise	RMR Indirect calorimetry J/kg/m Mean (SE)	Time of Day: 10:00 am Fasting: Overnight Temperature: 22 ± 1°C Pre-rest: 2 h Duration: 15 m	Follicular (days 6–10 of menstrual cycle) Luteal (days 21–25 of menstrual cycle)	RMR was significantly higher in the luteal phase than in the follicular phase.
1999 Li et al. [31]	Hong Kong	N = 19 Age range: 19–24 y Mean age: 21.3 y BMI: 19.5 kg/m^2^ Inclusion: No oral contraceptives; regular menstrual cycles; good health; no history eating disorders	RMR Indirect calorimetry kJ/d Mean (SD)	Time of Day: Morning Fasting: 10–12 h Temperature: NR Pre-rest: 30–40 m Duration: Average of last 10-m of 30-m period.	Mid-follicular (days 6–10 after menses onset) Mid-luteal (days 6–10 after ovulation confirmed by urinary luteinizing hormone)	RMR in mid-follicular phase was similar to that in the mid-luteal phase.
Year/Author	Origin	Sample	Measurements	Conditions	Time Points	Findings
1999Matsuo et al. [32]	Japan	N = 7 Age range: 18–20 y Mean age: NR BMI: 22.1 kg/m^2^ Inclusion: Physically active but no regular training at time of study	RMR EPOC Indirect calorimetry J/kg/m (RMR) L/6h (EPOC) Mean (SE)	Time of Day: 7:00 am Fasting: 12 h Temperature: 22 ± 1°C Pre-rest: 90 m (RMR) Exercise: 60 m (EPOC) Duration: 30 m (RMR); 6 h (EPOC)	Follicular (days 6–10 of menstrual cycle) Luteal (days 21–25 of menstrual cycle)	RMR and EPOC were significantly higher in the luteal phase versus the follicular phase.
1999 Paolisso et al. [33]	Italy	N = 16 Age range: NR Mean age: 26.1 y BMI: 21.1 kg/m^2^ Inclusion: Lean; not pregnant or breast-feeding; healthy; not diabetic or glucose intolerant; no known illness; normal menstrual cycles over past year; no oral contraceptives or other drugs; stable body weight and no dieting during previous 6 months; non-smokers; no physical training program during previous 6 months	BMR Indirect calorimetry kJ/m Mean (SE)	Time of Day: NR Fasting: 12–14 h Temperature: NR Pre-rest: NR Duration: NR	Follicular (days 4–7 after menstruation began) Periovulatory (day of luteinizing hormone surge ± 1 day) Luteal (days 23–27 after menstruation began)	No significant difference in BMR throughout the different phases of the menstrual cycle.
2000 Allen et al. [34]	United States	N = 21 Two groups: Smoking abstinence (n = 16); Smokers (n = 5) Age range: NR Mean age: 29 y BMI: 23.3 kg/m^2^ (smoking abstinence); 24.4 kg/m^2^ (smokers) Inclusion: NR	RMR Indirect calorimetry kcal/d Mean (SE)	Time of Day: 7:30–8:30 am Fasting: NR (taken before breakfast) Temperature: “Thermal neutral” Pre-rest: 15–30 m Duration: 15 m	Follicular (5 days after onset of menses) Late luteal (7–10 days before menses)	Average energy expenditure was higher during the late luteal phase compared to the follicular phase.
Year/Author	Origin	Sample	Measurements	Conditions	Time Points	Findings
2000 Fukuba et al. [35]	Japan	N = 5 Age range: 21–22 y Mean age: NR BMI: 20.3 kg/m^2^ Inclusion: Healthy; no regular exercise training; non-smokers; regular menstrual cycles	EPOC Two diet conditions (standard, restricted) Automatic gas analyzer L/7h Mean (SD)	Time of Day: 9:00 am Fasting: 1–5 h after breakfast (24-h diet controlled) Temperature: 25 ± 1°C Exercise: 60 m Duration: 7 h	Follicular (not defined) Luteal (not defined)	EPOC is not influenced by different phases of the menstrual cycle.
2001 Pelkman et al. [36]	United States	N = 20 Two groups: Contraceptive (n = 10); Placebo (n = 10) Age range: 21–34 y Mean age: 23.7 y BMI: 21.9 kg/m^2^ Inclusion: Regular menstrual cycles; no use of contraceptive hormones in previous year; no food restrictions; non-smokers; no medications known to affect appetite; not lactating or pregnant	RMR Indirect calorimetry Data NR	Time of Day: Before breakfast Fasting: 12 h Temperature: NR Pre-rest: 20 m Duration: 30 m	Follicular (3-5d before estimated ovulation) Luteal (6–10 d after positive ovulation test)	Subjects expended 4.3% more energy at rest in the luteal phase than in the follicular phase of the menstrual cycle.
2002 Horton et al. [37]	United States	N = 10 Age range: 18–39 y Mean age: 29 y BMI: 21.4 kg/m^2^ Inclusion: Regular menstrual cycle over the past year; habitually active but not highly trained competitive athletes	RMR Indirect calorimetry kJ/m Mean (SE)	Time of Day: 8:00 am Fasting: 12 h Temperature: NR Pre-rest: 30 m Duration: 15–20 m	Early follicular (days 4–6 after start of menstruation) Mid-follicular (after early follicular and before ovulation) Mid-luteal (after ovulation)	RMR tended to be greater (to a nonsignificant degree) in the mid-luteal than in the early follicular and mid-follicular phases of the menstrual cycle
Year/Author	Origin	Sample	Measurements	Conditions	Time Points	Findings
2002 Suh et al. [38]	United States	N = 8 recruited (Follicular n = 7; Luteal n = 5) Age range: 22–30 y Mean age: NR BMI: NR Inclusion: Nulliparous; normal menstrual flow for at least 6 months; no oral contraceptives; no changes in weight, exercise, or diet within last 6 months	RMR (post-prandial) Open circuit calorimetry kJ/m kcal/m Mean (SE)	Time of Day: NR Fasting: 3 h post standardized breakfast Temperature: NR Pre-rest: NR Duration: 15 m	Early follicular (days 3–9 of menstrual cycle) Luteal (days 18–24 of menstrual cycle or 4–9 days past luteinizing hormone surge confirmed with ovulation kits)	No difference between phases.
2005 Day et al. [39]	United States	N = 14 Age range: NR Mean age: 29 y BMI: 24.0 kg/m^2^ Inclusion: Eumenorrheic; non-smokers; no oral contraceptives or medications known to affect resting energy expenditure; healthy; resting heart rate of ≥ 50 bpm; normal serum thyroid stimulating hormone; normal treadmill stress test, and BMI ≤ 30.0 kg/m^2^	RMR Indirect calorimetry kcal/d kJ/d Mean (SE)	Time of Day: 6:00 am Fasting: 12 h Temperature: NR Pre-rest: ≈ 60 m Duration: 30 m	Early follicular (2–6 days after onset of menses) Mid-luteal (7–9 days after positive ovulation test)	RMR was higher in the mid-luteal phase than in the early follicular phase.
2005 Uranga et al. [40]	United States	N = 10 Age range: NR Mean age: 32 y BMI: 22.2 kg/m^2^ Inclusion: Non-obese; no oral contraceptives or medications; weight stable for at least 2 months	BMR Indirect calorimetry kcal/d Mean (SE)	Time of Day: 7:00 am Fasting: 12 h Temperature: NR Pre-rest: NR Duration: NR	Follicular (not defined) Luteal (not defined)	There were no significant differences between follicular and luteal BMR values.
Year/Author	Origin	Sample	Measurements	Conditions	Time Points	Findings
2006 Magkos et al. [41]	United States	N = 7 Age range: NR Mean age: 27 y BMI: 25.0 kg/m^2^ Inclusion: Normal fasting glucose; normal oral glucose tolerance; fasting plasma triglycerides < 100 mg/dl; good health; eumenorrheic; no oral contraceptives for ≥ 6 months; not pregnant; non-smokers; no medications known to affect lipid metabolism	RMR Indirect calorimeter kcal/m Mean (SE)	Time of Day: 9:00 am and 12:30 pm (values averaged) Fasting: 13–18 h Temperature: NR Pre-rest: NR Duration: 30 m each (values averaged)	Follicular (5–9 days after onset of menstruation) Luteal (2–6 days before onset of menstruation)	RMR was not different between the follicular phase and luteal phase.
2007 Smekal et al. [42]	Austria	N = 19 Age range: NR Mean age: 26.6 y BMI: 22.5 kg/m^2^ Inclusion: Regular menstrual cycle during previous 6 months; no oral contraceptives during previous 6 months; not pregnant	RMR (post-prandial) Open air spirometry mL/m mL/kg/m Mean (SD)	Time of Day: 9:00 am– 12:00 pm Fasting: 2 h (after standardized breakfast) Temperature: NR Pre-rest: NR Duration: NR	Follicular (low estradiol and low progesterone) Luteal (high estradiol and high progesterone)	No significant difference in the follicular phase versus the luteal phase.
2009 Hall et al. [43]	Australia	N = 15 Age range: NR Mean age: NR BMI: NR Inclusion: Aged 18–45 years; non-obese; good health; regular menstrual cycles for at least 6 month; no medications including oral contraceptives	BMR Indirect calorimetry kcal/24h Mean (SE)	Time of Day: 8:00 am Fasting: 8 h Temperature: NR Pre-rest: 10 m Duration: 20 m	Follicular (days 7–10 of menstrual cycle) Luteal (days 18–21 of menstrual cycle)	No significant change in BMR across the menstrual cycle.
Year/Author	Origin	Sample	Measurements	Conditions	Time Points	Findings
2011 Vaiksaar et al. [44]	Estonia	N = 11 Age range: NR Mean age: 18.4 y BMI: 22.7 kg/m^2^ Inclusion: Competitive rowers; menstrual cycle duration of 24–35 days for at least 6 months; no oral contraceptives for at least 6 months	RMR (post-prandial) Open circuit spirometry kcal/min Mean (SD)	Time of Day: 4:00–6:00 pm Fasting: 2 h (after standardized meal) Temperature: NR Pre-rest: NR Duration: NR	Follicular (days 7–11 from onset of menstruation) Luteal (days 18–22 from onset of menstruation)	No significant menstrual cycle phase effect was observed.
2015 Elliott et al. [45]	Singapore	N = 13 Age range: 21–28 y Mean age: 23.7 y BMI: 20.2 kg/m^2^ Inclusion: At least 3 preceding regular consecutive cycles of comparable lengths; not pregnant or lactating; no oral contraceptives, hormone supplements or medications that could affect menstrual cycle, metabolism, or body composition; non-smokers; no dietary restrictions	RMR Indirect calorimetry kJ/d Mean (SD)	Time of Day: NR Fasting: 8–12 h Temperature: NR Pre-rest: 10 m Duration: 20 m	Follicular (25–49% of normalized cycle) Luteal (51–100% of normalized cycle) [Menstrual cycle normalized between 0% (first day of menstrual period) to 50% (ovulation) to 100% (day before beginning of next menstrual cycle)]	No significant differences in RMR between phases of the menstrual cycle.
2015Matsuda-Nakamura et al. [46]	Japan	N = 8 Age range: NR Mean age: 22 y BMI: 21.3 kg/m^2^ Inclusion: Healthy; non-smokers; regular menstrual cycles; no oral contraception; spent most of the day in a room	RMR (post-prandial) Indirect calorimetry kJ/square meter (surface area)/h Mean (SE)	Time of Day: Morning Fasting: 2 h (after standardized breakfast) Temperature: 23.5°C Pre-rest: 40 m Duration: 80 m	Follicular (6–11 days after onset of menstrual flow) Luteal (4–10 days after elevation of basal body temperature >0.5°C indicating a change in menstrual phase)	RMR during cold exposure was not different in the follicular and luteal phases.

NR = not reported; RMR = resting metabolic rate; BMR = basal metabolic rate; SMR = sleeping metabolic rate; EPOC = excess post-exercise oxygen consumption

Inclusion criteria for participants varied widely. Having a regular menstrual cycle was most frequently reported (n = 20). The second most frequent inclusion criterion was no current use of oral contraceptives (n = 16), although one study included only women who used oral contraceptives. Ten studies enrolled non-smokers, while one study enrolled women who were both current and abstaining smokers. Five studies recruited only women who reported no regular exercise, and one study recruited regular exercisers (competitive rowers). Finally, 12 studies reported inclusion of women that were “healthy” or in “good health,” but there was no consistent definition of health used between studies.

### Metabolic measurement

Metabolic measurement was reported as RMR (n = 19), BMR (n = 9), SMR (n = 2), or EPOC (n = 2). The majority of studies (n = 19) reported measurement in a fasting state, six studies reported measurement of RMR, BMR, or EPOC after consumption of a standardized meal, and five studies did not provide a clear description. Indirect calorimetry (also reported as open and closed circuit calorimetry or spirometry, ventilated hood, and metabolic cart) was the most commonly reported method of measurement (n = 23), although use of Benedict-Roth apparatus (n = 2), Douglas bag (n = 2), metabolic chamber (n = 2), and automatic gas analyzer (n = 1) were also reported. The majority of studies reported the time of day when measurements were collected (n = 22), as well as the duration of the measurement period (n = 23). However, only nine studies reported the environmental temperature at the time of measurement.

### Risk of bias within studies

**Fig 2:**
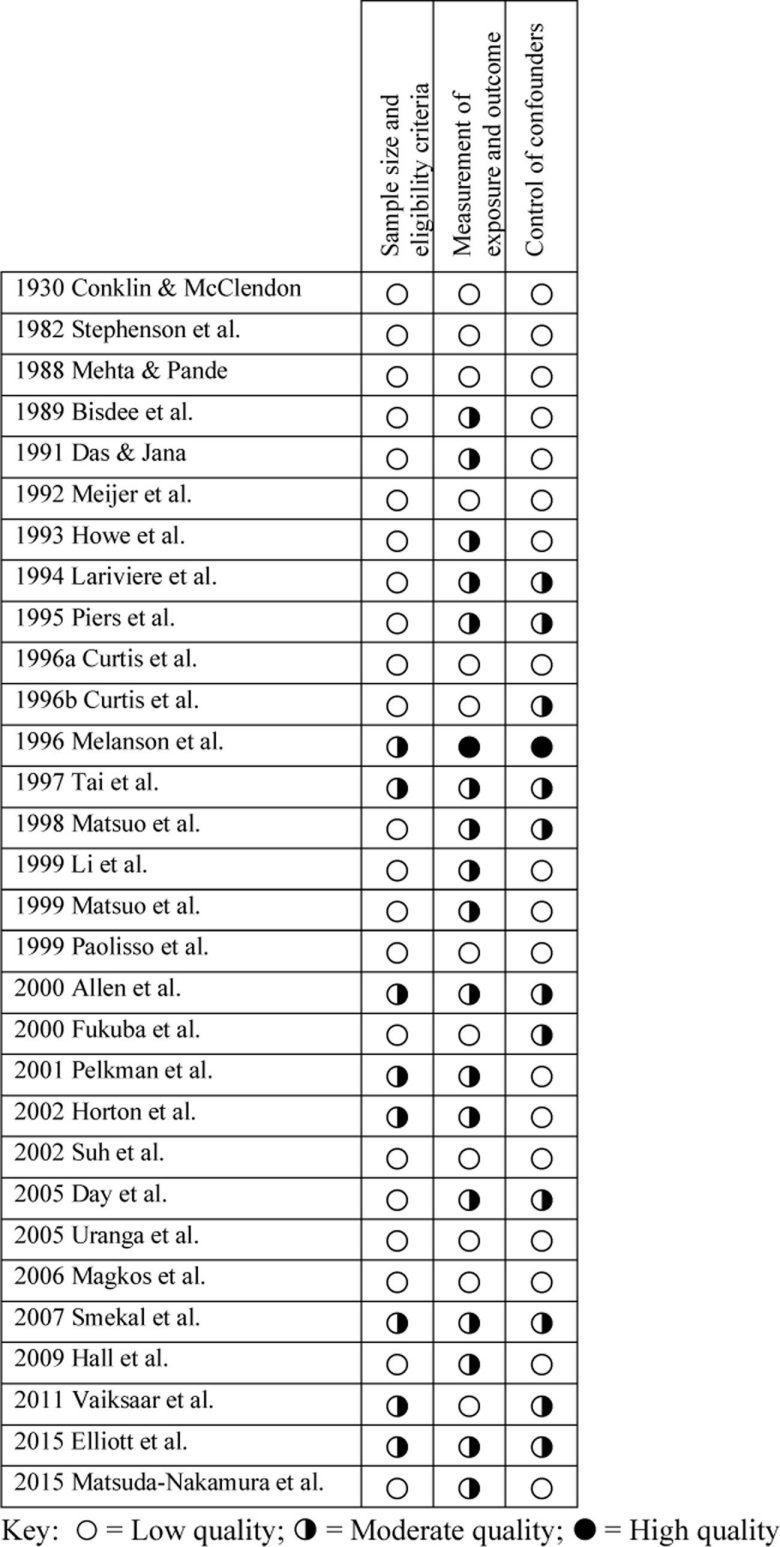
Quality assessment: Risk of bias in individual studies.

Quality ratings for individual studies are reported in Fig 2.

#### Sample size and eligibility criteria

Among the 30 studies included in the qualitative synthesis, risk of bias regarding sample selection, including size calculation for adequate power and clear inclusion/exclusion criteria, was universal. Quality was assessed as low for 22 studies and moderate for 8 studies. No studies were rated as high quality regarding sample selection. Specifically, while all but three studies reported inclusion criteria of some kind [10, 23, 27], only eight reported exclusion criteria [21, 27, 28, 34, 37, 42, 44, 45], and only one study reported power calculation for sample size [34].

#### Measurement of exposure and outcome

Risk of bias regarding measurement of both menstrual cycle and RMR was observed in all but one study [28], which was assessed as high quality. Otherwise, methodologically, 12 studies were assessed as low quality and 17 were assessed as moderate quality. Specifically, conditions for metabolic testing were reported clearly by the majority of studies, although seven studies did not provide sufficient detail to allow replication [19, 23, 26, 27, 38, 40, 44]. The timing of menstrual phases was also clearly defined in all but five studies [19, 26, 27, 35, 40], although definitions varied, with a range of 2–12 days after onset of menses for the follicular phase and a range of 16–30 days for the luteal phase. However, although two-thirds of the studies reported verification of menstrual phase, ten studies did not [19, 20, 23, 25–27, 40, 41, 44, 45]. Finally, no studies reported blinding of outcome assessors.

#### Control of confounders

Risk of bias regarding control of confounders was also substantial. In this area, 18 studies were assessed as low quality, 11 studies were moderate quality, and one study was assessed as high quality [28]. Specifically, only two studies controlled for caffeine intake [28, 34], only three controlled for alcohol intake [24, 28, 34], and only 13 studies controlled for smoking [25, 27–29, 33–36, 39, 41, 44–46]. Twelve studies controlled for pre-measurement exercise [24, 29, 30, 33–35, 37, 39, 40, 42, 44, 45], but only eight controlled for pre-measurement diet [30, 32, 34, 35, 38, 41, 42, 44]. Although 19 studies reported controlling for use of medications, eight of these considered only oral contraceptives [11, 25–27, 36, 38, 42, 46], and 11 studies did not report control for medications at all [10, 19–23, 30–32, 34, 35]. Finally, only 11 studies reported control of environmental temperature during measurement [10, 21, 25, 27–30, 32, 34, 35, 46].

### Results of individual studies

Overall, 47% of studies (n = 14) reported an increase in RMR favoring the luteal phase, while 53% (n = 16) reported no difference between phases. Of the four studies that did not report mean data for RMR and so were included in the qualitative synthesis only, two reported no effect of menstrual phase [20, 26], and two reported a greater RMR in the luteal phase [27, 36]. When compared by sample size, 50% (n = 8) of studies with samples sizes of 10 or less reported increased RMR in the luteal phase, while 50% (n = 8) reported no difference. In contrast, among larger studies with samples sizes greater than 10, only 43% (n = 6) reported a greater RMR during the luteal phase, while 57% (n = 8) reported no difference between phases. When compared by publication date, 59% (n = 10) of the studies published prior to the year 2000 reported greater RMR in the luteal phase, while 41% (n = 7) reported no difference. Alternately, among more recent studies published in the year 2000 or later, only 31% (n = 4) reported greater RMR during the luteal phase, compared to 69% (n = 9) that found no difference between phases.

### Meta-analysis

**Fig 3:**
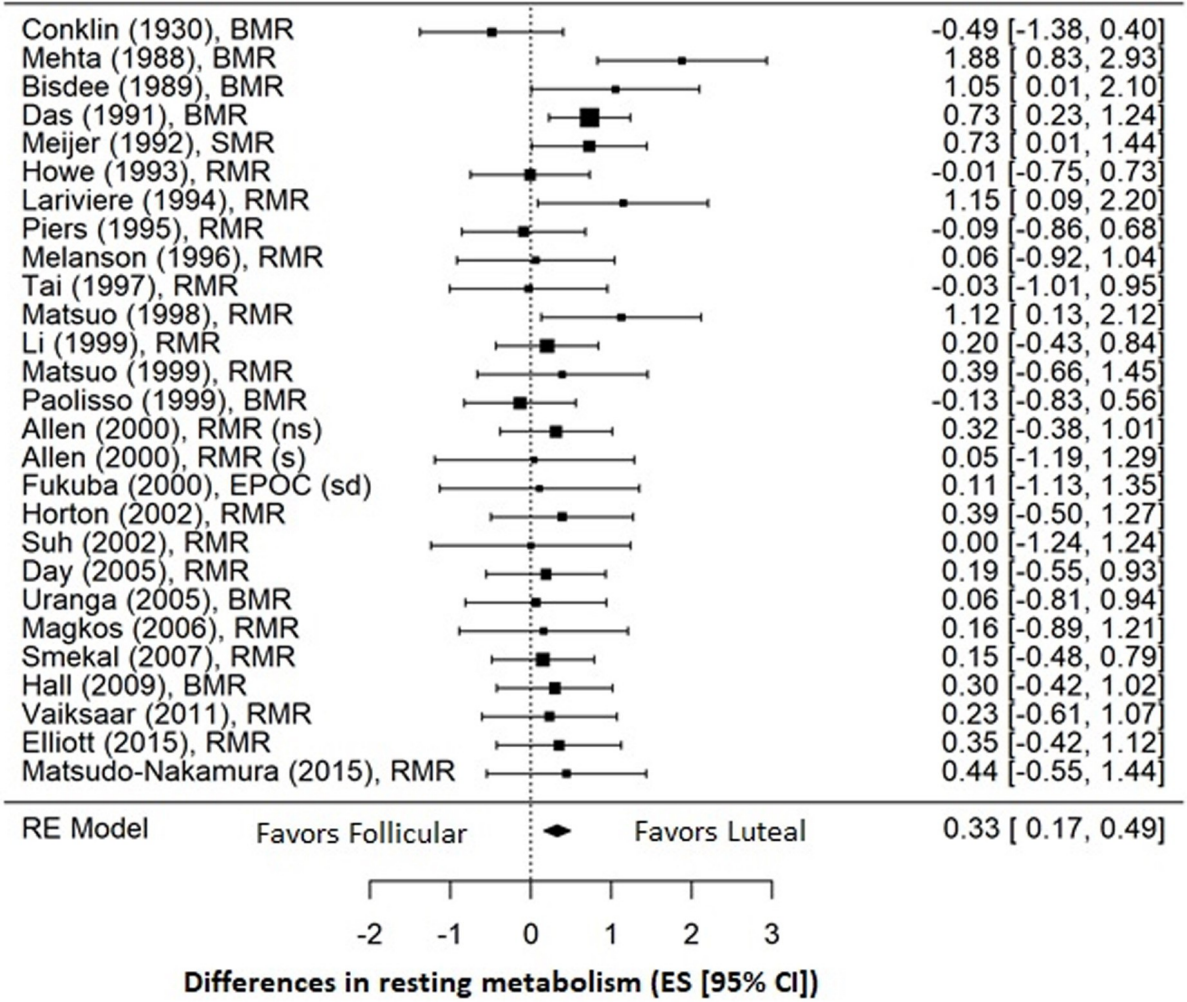
Forest plot of effect sizes for all 26 studies included in the meta-analysis. Studies are listed by first author and year of publication. The overall effect (ES = 0.33) was calculated using a random effects (RE) model and favors an increase in RMR during the luteal phase compared to the follicular phase. (ns) = non-smokers; (s) = smokers; (sd) = standard diet.

**Fig 4:**
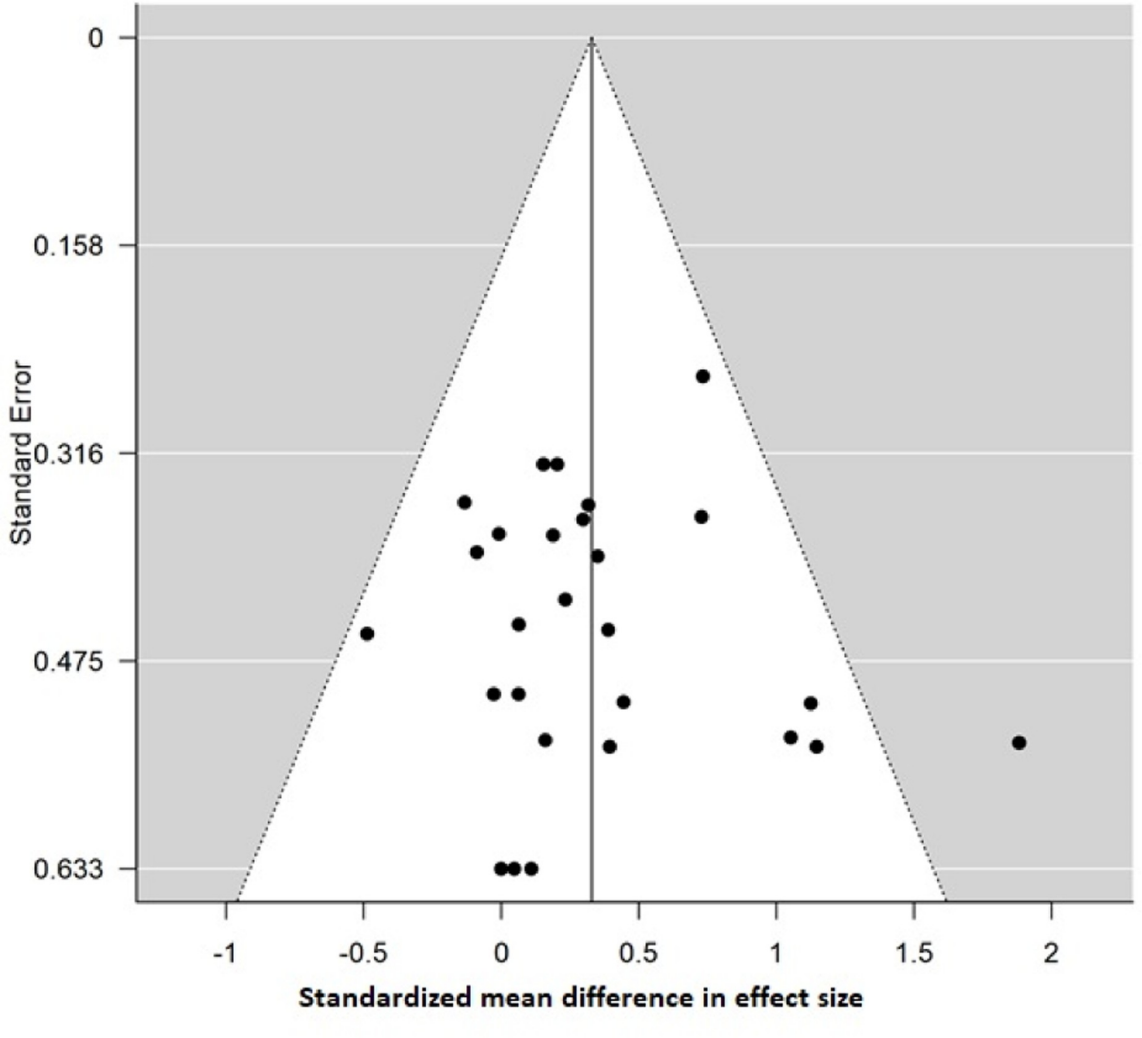
Funnel plot of effect sizes for all studies included in the meta-analysis. Egger’s test is non-significant (p = 0.721), indicating low risk of publication bias.

Pooled analysis of the 26 studies involving a total of 318 women for which quantitative data were available demonstrated a small effect favoring an increase in RMR during the luteal phase (ES = 0.33; 95% CI = 0.17, 0.49; *p* < 0.001) compared to the follicular phase (Fig 3). Overall, heterogeneity between studies was low (*I*^*2*^ = 3.8%). On visual inspection the funnel plot was symmetrical, and Egger’s test was non-significant (*p* = 0.721), indicating low risk of publication bias (Fig 4). Sensitivity analysis conducted by removing each study sequentially from the analysis demonstrated no individual effects on the overall findings (ES = 0.29–0.36; 95% CI = 0.12, 0.5; *p* < 0.001), and removal of the two studies published by the same author [30, 32] also had no effect on the pooled analysis (ES = 0.31; 95% CI = 0.14, 0.47; *p* < 0.001) that continued to favor increased RMR during the luteal phase.

**Fig 5:**
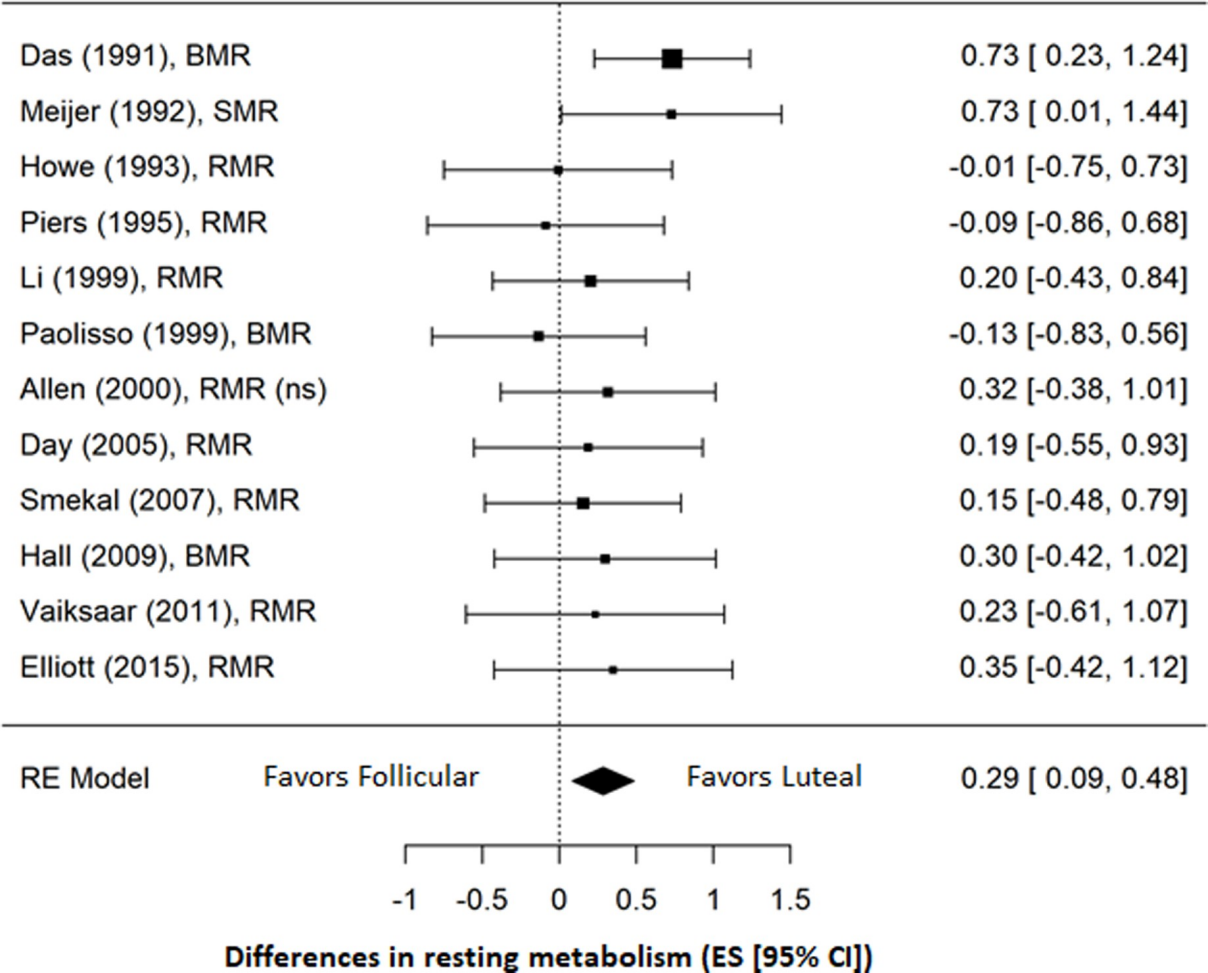
Forrest plot of sub-group analysis of studies with samples of more than 10 participants. (ns) = non-smokers.

Sub-group analysis conducted for 12 studies reporting a sample size of more than 10 participants resulted in a slightly smaller, but still significant effect that continued to favor increased RMR during the luteal phase (ES = 0.29; 95% CI = 0.09, 0.48; *p* = 0.005) (Fig 5). There was no evidence of heterogeneity among studies (*I*^*2*^ = 0.0%), and risk of publication bias remained low based on Egger’s test (*p* = 0.122).

**Fig 6:**
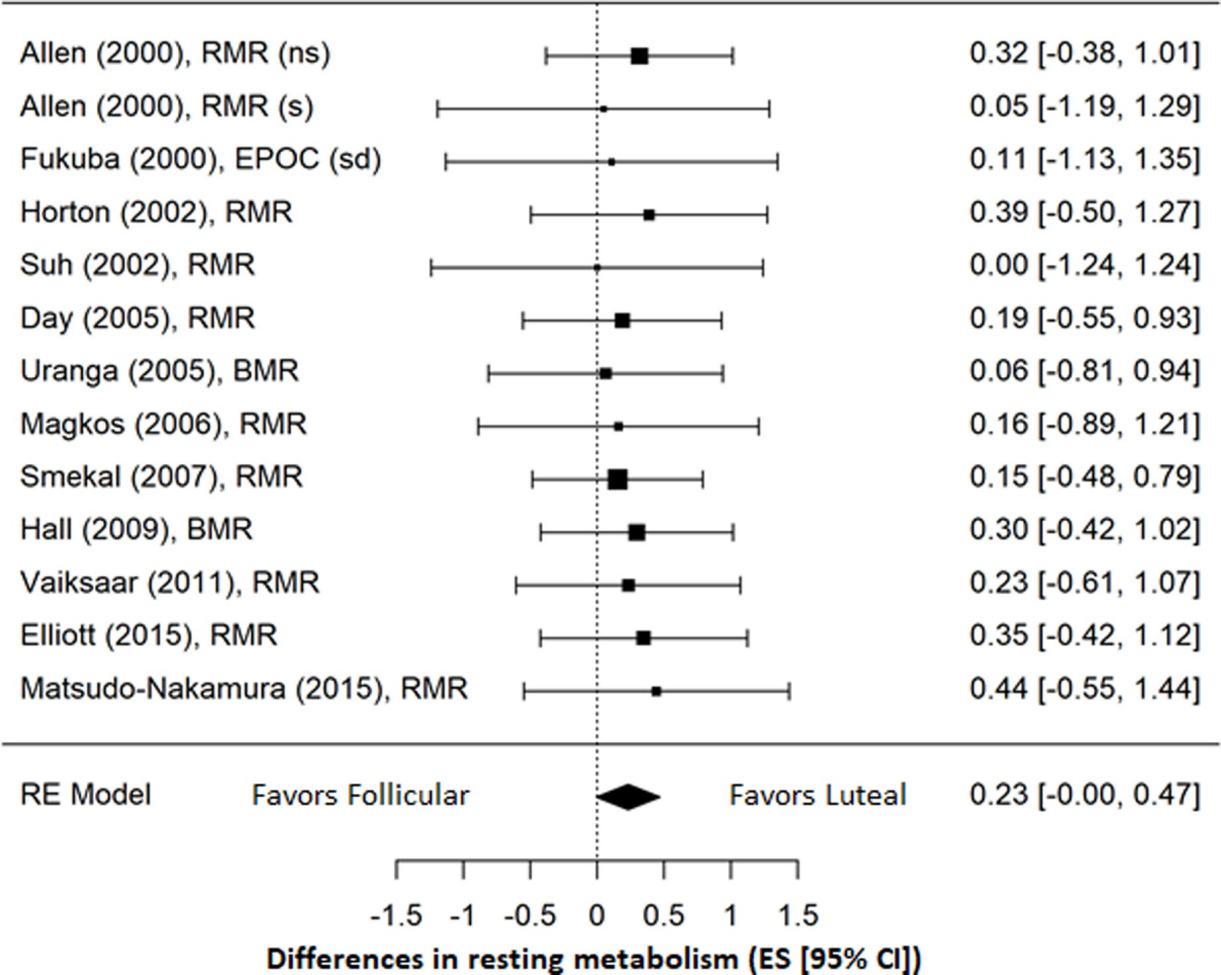
Forrest plot of sub-group analysis of studies published in 2000 and after. (ns) = non-smokers; (s) = smokers; (sd) = standard diet.

Sub-group analysis conducted for 12 studies published in the year 2000 or after (Fig 6) resulted in an even smaller and no longer significant effect of the menstrual cycle on RMR (ES = 0.23; 95% CI = -0.00, 0.47; *p* = 0.055). There continued to be no evidence of heterogeneity among studies (*I*^*2*^ = 0.0%) and risk of publication bias remained low based on Egger’s test (*p* = 0.745).

## Discussion

The evidence synthesized for this systematic review and meta-analysis spans a period of approximately 90 years. The principle finding was that when all studies were considered the menstrual cycle exerted a small, but statistically significant effect on RMR in women. Specifically, RMR was found to be greater during the luteal phase compared to the follicular phase. However, when we included only larger studies of more than 10 women the effect of menstrual cycle was slightly reduced, and when we considered only more recent studies published since 2000 the effect was even smaller and no longer significant.

Unfortunately, due to methodological differences among studies, we could not directly analyze the SMD in metabolic rate between the follicular and luteal phases of the menstrual cycle. We were precluded by individual study differences in units of measurement, differences in calculating metabolic rate (oxygen consumption versus energy expenditure), differences in measurement devices, and differences in resting conditions (RMR, BMR, SMR, EPOC). For that reason, we restricted our meta-analysis to pooled effect sizes, which does not provide an estimate of the actual difference in metabolic rate that could be anticipated across the menstrual cycle.

We believe it is important to determine the actual effect of the menstrual cycle due to the burden placed on researchers and participants in order to control for it. Possibly because of this burden, neither verification of nor control for menstrual cycle phase is universally reported by researchers measuring metabolism in young women. Recently published studies that include cross-sectional comparisons of RMR [47], training related changes in RMR [48], and validation of prediction equations for RMR [49] have either failed to report control or chosen not to control for the potential influence of the menstrual cycle on RMR. This inconsistency makes it difficult to evaluate the quality and impact of contemporary research findings and to accurately replicate study designs. If indeed menstrual cycle phases exert a trivial or null effect on metabolism, this should be clearly established in order to minimize any concerns regarding the validity of research findings.

Our qualitative synthesis also identified methodologic problems in individual studies. The majority of studies were low quality and based on small sample sizes. Ideally, to detect a medium effect between two independent sample means, a sample size of 64 is needed [16]. In contrast, the largest sample size among the studies included in our analysis was 32 [22], which is only half of what would be required. Consistent with our sub-group analysis that found a reduced effect when only larger studies were included, it seems possible that small sample sizes may have influenced the overall effect, and adequately powered studies with larger samples may determine that menstrual phases have a null effect on RMR.

Differences in the technology of the measurement devices may also have influenced our overall findings. When our analysis was limited to more recently published studies, a statistically significant effect of the menstrual cycle on RMR was no longer observed. Although we can find no evidence regarding the comparable accuracy of newer versus older technologies, it is possible that measurement has become more accurate over time with subtle improvements to measurement devices provided by the manufacturers. Also, more recent studies may have controlled more carefully for potential confounders. We acknowledge the paucity of detail provided by some of the studies, and in fact, a consistent pattern of methodological problems in menstrual cycle research has recently been identified, including small sample sizes and inadequate verification of menstrual phase at the time of testing [50].

Previously published recommendations for measurement of RMR with indirect calorimetry [51, 52] have addressed some of the methodological problems identified in our current review. These include guidelines for control of environmental temperature; physical activity; use of alcohol, nicotine, and caffeine; pre-measurement fasting; pre-measurement rest; and collection time [51, 52]. Due to incomplete reporting of methodologies among the studies synthesized in our analysis, it is unclear whether their designs controlled for all of these factors, and so the influence of potential confounders cannot be excluded. Our findings support the need for future research with larger sample sizes and complete reporting of methodologies, as well as studies comparing different gas collection devices, all of which have previously been recommended [51, 52].

### Strengths and limitations

We recognize that there were limitations to our meta-analysis. Our search strategy was limited to English-language publications only, and so we may not have identified all appropriate studies for inclusion in our meta-analysis. Furthermore, as previously discussed, the majority of studies were of low quality. Especially among the older studies, data reporting did not meet current expectations and methodology was not adequately described. Nevertheless, we used sub-group analyses to compensate for methodological inadequacies and believe that the effect sizes generated reflect the appropriateness of our approach. In addition, the single-group repeated measures design of the studies in our analysis is a strength, in that it controlled for the individual differences that are characteristic of other two-group comparison designs.

Outside of general methodological concerns, imprecision regarding measurement of menstrual phase is a unique limitation of the studies included in our analysis. Although the average menstrual cycle length is 29 days, individual variability can exceed 7 days [53]. Over and above the differences in the definition of the two menstrual phases, one-third of the studies in our analysis failed to confirm menstrual status other than through self-report of menses. It is therefore possible that the values reported for the follicular and luteal phases were not true values.

## Conclusion

Until larger and better designed studies are available, based on our current findings, researchers should be aware of the potential confounding influence of the menstrual cycle and control for it by testing consistently in one phase of the cycle when measuring RMR in pre-menopausal women. This is especially important when conducting sequential measurements. Furthermore, when disseminating research results, researchers should conscientiously provide a detailed report of their methodology that allows accurate replication of their design.

## Supporting information

S1 Checklist PRISMA 2009 checklist. (DOCX)

S1 Data (CSV)
